# Impact of pregnancy and passive smoking on lung function among rural South Indian women

**DOI:** 10.6026/973206300212442

**Published:** 2025-08-31

**Authors:** Mythili Bai K., Madhavrao Chavan, Santenna Chenchula, Padmavathi R., Anandhalakshmi A., Devika T.

**Affiliations:** 1 Department of Physiology, Government Siddhartha Medical College, Vijayawada, Andhra Pradesh, India; 2 Department of Pharmacology, All India Institute of Medical Sciences (AIIMS), Mangalagiri, Andhra Pradesh, India; 3 Department of Pharmacology, All India Institute of Medical Sciences (AIIMS), Bhopal, Madhya Pradesh, India; 4 Department of Pharmacology, Mediciti Institute of Medical Sciences, Hyderabad, Telangana, India; 5 Department of Pharmacology, Madras Medical College, Chennai, Tamil Nadu, India; 6 Department of Pharmacology, Guntur Medical College, Guntur, Andhra Pradesh, India

**Keywords:** Pregnancy, environmental tobacco smoke, pulmonary function tests, spirometry, rural women, small airway obstruction

## Abstract

Pregnancy induces physiological changes that influence pulmonary function, while environmental tobacco smoke (ETS) exposure is a known respiratory hazard. Therefore, it is of interest to evaluate the independent and combined effects of pregnancy and ETS exposure on pulmonary function tests (PFTs) in 200 rural South Indian women divided into four equal groups. Pulmonary parameters were measured using computerised spirometry and analysed using one-way ANOVA. Results showed that ETS exposure significantly impaired lung function, with the greatest decline observed in pregnant women exposed to ETS. Thus, we show the importance of minimising ETS exposure during pregnancy to protect maternal and fetal respiratory health.

## Background:

Pregnancy involves complex physiological adaptations across various systems, including the respiratory tract, which may influence the clinical interpretation of respiratory symptoms [1, 2]. Among the most notable are changes in the respiratory system. As pregnancy progresses, particularly in the later trimesters, the growing fetus displaces the diaphragm, thereby altering lung volumes and respiratory parameters [3]. Additional chest wall modifications include an increase in the lower chest diameter, expansion of the rib cage subcostal angle and relaxation of costal and pelvic ligaments due to the hormone relaxin. These anatomical shifts, such as an increase of 2 cm in chest wall diameters and a subcostal angle expansion from 68.5° to 103.5°, help accommodate the enlarging uterus but may also impact pulmonary function during pregnancy [4]. Globally, nearly one-third of the population smokes, and tobacco use accounts for approximately 4 to 5 million deaths annually [5]. In many parts of the world, cigarette smoking remains a major, yet preventable, cause of morbidity and mortality, particularly in the context of adverse pregnancy outcomes such as prematurity [5]. Environmental tobacco smoke (ETS) exposure, or passive smoking, is now recognised as a significant public health hazard with well-documented risks not only to children and women but also to the developing fetus [6]. Alarmingly, passive smokers incur health risks comparable to active smokers [4]. ETS is a major source of indoor air pollution and contains numerous toxic substances, including lead, cadmium, nicotine and polycyclic aromatic hydrocarbons [7]. Exposure prevalence is exceptionally high in Southeast Asia among pregnant women and children [8], with rural populations facing elevated risks due to overcrowding and poor ventilation. Therefore, it is of interest to quantify pregnancy-associated changes in pulmonary function tests (PFTs), to assess the independent impact of ETS exposure on PFTs, and to evaluate the combined effects of pregnancy and ETS exposure on respiratory parameters in rural South Indian women from Kanyakumari District, Tamilnadu, India.

## Methods:

## Study design and setting:

This descriptive, comparative study was conducted in the Department of Physiology in collaboration with the Department of Obstetrics and Gynaecology at Sree Mookambika Institute of Medical Sciences, Kulasekharam (Kanyakumari District), Tamil Nadu, India.

## Sample size determination:

The sample size was determined based on findings from previous literature evaluating the effects of pregnancy and smoking on pulmonary function tests (PFTs). Phatak *et al.* studied 50 pregnant women and 50 non-pregnant controls to examine antenatal changes in PFTs [9]. Ritesh *et al.* compared PEFR and MVV in smokers and non-smokers, while Gupta *et al.* analysed differences in PFTs among ETS-exposed and non-exposed women, each group comprising 50 participants [6, 10]. Similarly, Teli *et al.* conducted a cross-sectional study with 200 women divided into four groups (n = 50 per group) across different trimesters of pregnancy and matched controls [11]. Based on these precedents, our study enrolled a total of 200 women, equally divided into four groups of 50 participants each.

## Study groups:

Participants were categorised into the following four groups:

[1] Group I: Non-pregnant women not exposed to environmental tobacco smoke (ETS)

[2] Group II: Non-pregnant women exposed to ETS

[3] Group III: Pregnant women (second or third trimester) not exposed to ETS

[4] Group IV: Pregnant women (second or third trimester) exposed to ETS

## Eligibility criteria:

Women aged 20 to 35 years residing in rural areas of Kanyakumari district with haemoglobin levels greater than 10 g/dL were eligible for inclusion. Pregnant women were eligible for Groups III and IV only if they were in their second or third trimester. ETS exposure, defined as the presence of active smokers (≥10 cigarettes or beedis per day) in the household or workplace for a minimum of six months before recruitment, was required for Groups II and IV. The Exclusion criteria included multiple pregnancies, structural deformities of the chest or spine, and a history of chronic use of medications that could alter bronchial tone, such as beta-adrenergic agonists/antagonists, phosphodiesterase inhibitors, cholinergic agents, histamine-releasing drugs, and NSAIDs. Participants with a known history of respiratory or cardiovascular disorders were also excluded.

## Instruments and Measurements:

Pulmonary function was assessed using the Spiro Excel spirometer (Medicaid Systems, Chandigarh, India), a computerized instrument capable of measuring 34 different parameters. The device includes a digital turbine, USB interface, nose clips, and adult/child mouthpieces. Height was measured using a standard measuring tape (Voadham Tapes, Delhi), body weight with a calibrated digital scale (Goldtech Sachdeva Traders, Gurgaon), and blood pressure using a mercury sphygmomanometer (Lifeline, Bombay). Hemoglobin was estimated via Drabkin's cyanmethemoglobin method in the central laboratory of the institution.

## Ethical considerations:

Ethical approval was obtained from the Institutional Human Ethics Committee (IHEC) of Sree Mookambika Institute of Medical Sciences, Kulasekharam (Ref. No. SMIMS/IHEC/2012/A1/03). Informed written consent was obtained from each participant before enrollment.

## Study procedure:

Participants were recruited from the antenatal clinic (for pregnant women) and outpatient departments (for non-pregnant controls). Eligible women were grouped based on pregnancy status and ETS exposure. Demographic details including age, height, weight, hemoglobin, and blood pressure were recorded on a predesigned case record form. All pulmonary function testing was conducted between 10:00 AM and 12:00 PM to avoid the confounding effects of diurnal variation. Before the actual test, each subject was given a thorough explanation and a live demonstration of each maneuver. Tests were conducted in a seated position with feet flat on the floor, and each maneuver was repeated three times at five-minute intervals. The average of the three measurements was used for final analysis.

## Pulmonary function testing maneuvers:

[1] Maneuver 1 was performed to assess Forced Vital Capacity (FVC), Forced Expiratory Volume in 1 second (FEV1), FEV1/FVC ratio, Peak Expiratory Flow Rate (PEFR), Peak Inspiratory Flow Rate (PIFR), and Forced Expiratory Flow at 25%, 50%, 75%, and 25-75% of expiration (FEF25%, FEF50%, FEF75%, FEF25-75%). Participants were instructed to take a deep breath, seal the mouthpiece tightly between their lips, and exhale forcefully with the nose clipped.

[2] Maneuver 2 was used to evaluate Tidal Volume (TV), Expiratory Reserve Volume (ERV), and Inspiratory Reserve Volume (IRV). Participants first performed normal tidal breathing, followed by a full expiration and inspiration, and then resumed normal breathing.

[3] Maneuver 3 was conducted to assess Maximum Voluntary Ventilation (MVV). Participants were instructed to breathe deeply and rapidly at approximately 30 breaths per minute through the mouthpiece with the nose clipped.

## Statistical analysis:

All data were entered using Microsoft Excel 365 and analyzed using GraphPad Prism version 10.1.2 (GraphPad Software Inc., San Diego, CA, USA). Descriptive statistics were presented as mean ± standard deviation (SD) in tables and as mean ± standard error (SE) in bar diagrams. Group comparisons were performed using one-way analysis of variance (ANOVA) followed by Bonferroni's post hoc test. A p-value of less than 0.05 was considered statistically significant.

## Results:

A total of 200 participants were included in the study after applying the inclusion and exclusion criteria. Participants were equally divided into four groups (n=50 each). Baseline characteristics such as age, height, weight, haemoglobin levels, blood pressure, duration of ETS exposure, and gestational age were comparable across groups. These details are provided in Supplementary Table 1 (see PDF). Forced Vital Capacity (FVC) was significantly lower in Group II and Group IV compared to Group I and Group III (P<0.001), indicating a deleterious impact of ETS exposure. Similarly, Forced Expiratory Volume in 1 second (FEV1) was significantly reduced in ETS-exposed groups (Groups II and IV) when compared with their respective non-exposed counterparts (P<0.001). The FEV1/FVC ratio was also significantly decreased in Group II and Group IV when compared to Groups I and III (P<0.001), with a further decline noted in Group IV relative to Group II (P<0.001). Conversely, Group III exhibited a statistically significant increase in FEV1/FVC compared to Group I (P<0.05), suggestive of adaptive physiological changes in pregnancy. Statistically significant reductions in PEFR and PIFR were seen in ETS-exposed groups, particularly in Group IV, when compared to Groups I and III (P<0.001). Mid-expiratory flows, including FEF25-75%, FEF25%, FEF50%, and FEF75%, were consistently lower in Groups II and IV compared to Groups I and III (P<0.001), highlighting small airway involvement due to ETS exposure. Expiratory Reserve Volume (ERV) was significantly reduced in Groups III and IV compared to Group I (P<0.05 and P<0.001, respectively). Group IV also had lower ERV compared to both Group II (P<0.001) and Group III (P<0.01). However, no statistically significant difference was observed in Inspiratory Reserve Volume (IRV) among the groups (P>0.05). Tidal Volume (TV) was significantly higher in Groups III and IV compared to Groups I and II (P<0.001), reflecting physiological hyperventilation during pregnancy. Maximum Voluntary Ventilation (MVV) showed a decreasing trend in Group IV, but without statistical significance. Pairwise group comparisons further highlighted the impact of ETS exposure. Group II had significantly lower values for FEV1, PEFR, FEF25-75%, and FEV1/FVC compared to Group I (P<0.001), detailed in Supplementary Table S1 (see PDF). Likewise, Group IV demonstrated lower values for the same parameters compared to Group III (P<0.001), as presented in Supplementary Table S2 (see PDF). Group III, when compared to Group I, had significantly increased TV and FEV1/FVC and significantly reduced ERV (P<0.001 and P<0.05, respectively). Although IRV appeared higher in Group III, this increase was not statistically significant (P>0.05). This comparative data is shown in Supplementary Tables S3 (see PDF) & S4 (see PDF).

## Discussion:

The present study aimed to evaluate the effects of pregnancy and environmental tobacco smoke (ETS) exposure on pulmonary function tests (PFTs) in women from rural South India, specifically in the Kanyakumari District, where ETS exposure remains highly prevalent [12, 6]. A total of 200 participants were categorized into four groups of 50 each: Group I (non-pregnant women not exposed to ETS), Group II (non-pregnant women exposed to ETS), Group III (pregnant women not exposed to ETS), and Group IV (pregnant women exposed to ETS). Our study showed a significant decline in FVC, FEV1, FEV1/FVC, PEFR, PIFR, FEF25-75%, FEF25%, FEF50%, and FEF75% in ETS-exposed subjects. The decline was highest in pregnant women exposed to ETS (Group IV), followed by non-pregnant women exposed to ETS (Group II). We observed a significant increase in Tidal Volume (TV) in both Group III and Group IV compared to Groups I and II. However, the increase was more pronounced in Group III, though not statistically significant compared to Group IV. The rise in TV among pregnant women may be attributed to increased ventilation induced by progesterone hormone-mediated stimulation of respiration [13-14]. This study also found a significant decline in Expiratory Reserve Volume (ERV) in Group III compared to Group I, consistent with findings reported by Gazioglu *et al.* [15]. The reduction in ERV was even more marked in Group IV compared to Groups III, I, and II. A slight, non-significant increase in Inspiratory Reserve Volume (IRV) was observed in pregnant groups compared to non-pregnant groups, consistent with the findings of Gazioglu *et al.* [15]. Similarly, there was a non-significant reduction in Maximum Voluntary Ventilation (MVV) in Groups II, III, and IV compared to Group I. The highest decline in MVV was found in Group IV, possibly due to the combined obstructive and restrictive effects of ETS exposure and pregnancy.

Significant decreases in FVC and FEV1 in Groups IV and II compared to the other groups were in line with earlier studies [16]. Similarly, Gallotti *et al.* also reported a significant decline in FVC among ETS-exposed individuals [17]. Gupta *et al.* also found a significant decrease in FEV1 in ETS-exposed individuals, which aligns with our findings [6]. The reduction in FVC and FEV1 suggests airway obstruction [6, 13]. In our study, the decline in these parameters may be due to airway obstruction resulting from ETS exposure. A slight, non-significant reduction in FVC was observed in Group III compared to Group I, similar to results by Teli *et al.* who reported a significant reduction [11]. However, the reduction in Group IV compared to Group I in our study may reflect the additive effect of ETS exposure during pregnancy. FEF25-75% and FEF75% are considered early indicators of small airway obstruction [18]. In our study, these parameters were significantly decreased in ETS-exposed groups (Groups II and IV) compared to non-exposed groups (Groups I and III), indicating a significant impact of ETS on small airway airflow obstruction. These results are consistent with previous studies [16, 17 18-19], which also reported a significant decline in FEF25-75% and FEF75% among ETS-exposed female flight attendants. Other PFT parameters-PEF, PIFR, FEF25%, and FEF50%-were significantly reduced in ETS-exposed groups (Groups II and IV) compared to non-exposed groups (Groups I and III). These reductions suggest airway narrowing and increased airflow resistance, likely due to decreased lung elastic recoil [5- 6, 18]. Similar findings have been reported by Harirah *et al.*, Sunyal *et al.* and Teli *et al.* who also found a significant decrease in PEFR among pregnant women, aligning with our study's observation of a slight, though non-significant, reduction in Group III compared to Group I [20-21, 11]. The FEV1/FVC ratio was decreased in ETS-exposed groups (Groups II and IV) compared to non-exposed groups (Groups I and III), supporting findings by Bhargava *et al.* This reduction signifies an obstructive lung pattern [18- 19]. In contrast, Group III showed a significant increase in FEV1/FVC compared to other groups, which may reflect physiological restriction due to diaphragmatic elevation during pregnancy [18]. The lower ratio in Group IV may suggest a dominant obstructive effect of ETS that overrides pregnancy-related changes.

Group III showed non-significant reductions in several PFT parameters (FVC, PEFR, PIFR, FEF25-75%, FEF25%, FEF50%, FEF75%) compared to Group I, which may reflect the restrictive influence of anatomical and physiological changes during pregnancy [18]. However, significant reductions in these parameters in Group IV compared to Group III highlight the additive effect of ETS exposure during pregnancy. Progesterone levels remain elevated throughout pregnancy and contribute to respiratory stimulation and bronchodilation via prostaglandin-mediated smooth muscle relaxation [13-14, 22]. Despite this, significant reductions in FVC, FEV1, FEV1/FVC, PEFR, PIFR, FEF25-75 %, FEF25%, FEF50%, and FEF75% in Group IV suggest that ETS exposure may override progesterone's protective respiratory effects. In contrast to our findings, a study by Tredaniel *et al.* did not observe significant changes in pulmonary function between ETS-exposed and non-exposed individuals [16]. We controlled for several confounding variables, including age, height, weight, hemoglobin levels, gestational age, and ETS exposure duration. All groups were matched for these factors. Hemoglobin concentration was standardized above 10 g/dL, as even borderline variations can affect pulmonary function [2]. All PFTs were conducted between 10:00 AM and 12:00 PM to minimize diurnal variability [23]. Nevertheless, some limitations exist. We did not control for the type of household fuel (*e.g.*, wood, coal, kerosene), daily ETS exposure duration, or indoor air pollution. Nor did we quantify ETS exposure biochemically using cotinine levels in saliva, serum, or urine [24-25]. Additionally, some participants may have been exposed to more beedi smoke than cigarette smoke, which could confound results as beedis contain more nicotine [6]. The decline in PFT parameters among ETS-exposed groups in our study supports evidence that passive smokers are at risk of respiratory impairment similar to that of active smokers [19, 26-27]. From this present study, it is evident that pregnancy alters pulmonary function through physiological and anatomical adaptations, and ETS exposure further exacerbates pulmonary compromise by inducing obstructive changes, particularly in small airways. The combination of pregnancy and ETS exposure poses the greatest risk. Therefore, it is imperative to raise awareness among women about the harmful effects of ETS exposure, especially during pregnancy, for the well-being of both mother and fetus.

## Conclusion:

Pregnancy induced physiological changes in lung function, notably increasing tidal volume and FEV1/FVC ratio while reducing ERV. Environmental tobacco smoke (ETS) exposure significantly impaired pulmonary function across multiple parameters, with the greatest decline seen in pregnant women exposed to ETS. Thus, we show the urgent need to reduce ETS exposure during pregnancy to protect maternal and fetal respiratory health.

## Funding:

None

## Ethical approval:

The study was approved by the Institutional Human Ethics Committee of Sree Mookambika Institute of Medical Sciences, Kulasekharam, Tamil Nadu (Ref. No. SMIMS/IHEC/2012/A1/03). Written informed consent was obtained from all participants before enrollment.

## Author contributions:

MBK: Study design, data acquisition, and drafting the manuscript; MC: Conceptual support, data interpretation, and critical revision; SC: Data analysis, manuscript preparation and final approval; PR: Literature review and referencing; AA: Data validation and statistical review; DT: Formatting, proofreading, and table/figure preparation. All authors have read and approved the final version of the manuscript.
